# Effect of the *Lippia alba* (Mill.) N.E. Brown essential oil and its main constituents, citral and limonene, on the tracheal smooth muscle of rats

**DOI:** 10.1016/j.btre.2017.12.002

**Published:** 2017-12-06

**Authors:** Poliana M.M. Carvalho, Cícero A.F. Macêdo, Tiago F. Ribeiro, Andressa A. Silva, Renata E.R. Da Silva, Luís P. de Morais, Marta R. Kerntopf, Irwin R.A. Menezes, Roseli Barbosa

**Affiliations:** a Physiopharmacology of Excitability Cell Laboratory, Department of Chemical Biology Regional University of Cariri, Campus of Pimenta, 63105-010, Crato, CE, Brazil; b Pharmacology of Natural Products Laboratory, Regional University of Cariri-Campus of Pimenta, 63105-010, Crato, CE, Brazil; c Pharmacology and Molecular Chemistry Laboratory, Department of Chemical Biology, Regional University of Cariri-Campus of Pimenta, 63105-010, Crato, CE, Brazil

**Keywords:** *Lippia alba*, Essential oil, Citral, Smooth muscle, Limonene

## Abstract

The *Lippia alba* (Mill.) N.E. Brown (Verbenaceae) species, has effects sedative, analgesic and spasmolytic properties. This study had as its main objective to evaluate the essential oil of *L. alba* (EOLa) effect and that of its main constituents, citral and limonene, over tracheal smooth muscle from Wistar rats. EOLa, citral and limonene promoted relaxation of tracheal smooth muscle in contractions induced by potassium (60 mM K^+^), presenting an EC_50_ of 148 ± 7 μg/mL for the EOLa, 136 ± 7 μg/mL for citral and 581 ± 7 μg/mL for limonene. In contractions induced by Acetylcholine (Ach; 10 μM) the EC_50_ for the EOLa and citral were of 731 ± 5 μg/mL and 795 ± 9 μg/mL, respectively. In preparations pre-incubated with 1000 μg/mL of the EOLa and citral, both agents were found to block the influx of BaCl_2_ by VOCCs. This study demonstrated that the EOLa and its main component citral present antispasmodic effect over tracheal smooth muscle of rats.

## Highlights

• EOLa and citral promoted relaxation of tracheal smooth muscles in contractions induced by potassium and by acetylcholine.
• The effect of EOLa and citral on tracheal smooth muscles is mediate by reduction or absence of Ca^2+^ influx.
• Limonene less potent than EOLa and citral and does not act through voltage-operated cationic Ca^2+^ influx through channels.

## 1 Introduction

The species *Lippia alba* (Mill.) N. E. Brown (Verbenaceae) is an aromatic shrub native to South America, Central America and Africa. In Brasil, *L. alba* is known popularly as “lemon balm”, “false melissa” and “field rosemary” and it is readily used in the form of infusion or decoction of the leaves for the treatment of cold, bronchitis, cough, asthma and intestinal disorders [1]. Pharmacological activities have also been verified for the species: antioxidant, sedative [2], anxiolytic [3] and antispasmodic [4].

Citral and limonene are the main components of the essential oil of *L. alba*. Citral is a natural mixture of aldeidos, being the geranial ((E)-3.7-dimethyl-2.6-octadienal) and the Neral ((Z)-3.7-dimethyl-2.6-octadienal), where the chemotype under study is classified as chemotype II (MATO et al., 1996) [5]. Pharmacological studies attribute to citral the following activities: acaricide, insecticide and anxiolytic, [6] as well as sedative [7]. Limonene, (*R*)4-isoprenyl-1-methyl-cyclohexene, is a monocyclic monoterpene present in the structure of many plants such as *Mentha* spp. and citrus plants. Pharmacologically, activities such as: antifungal, antibacterial, antitumor, acaricide, insecticide and repellent activities, have been attributed to limonene [[8], [9], [10], [11], [12]].

Currently, the pharmaceutical industry have been searching for substances in natural products which can improve the treatment of many diseases, among which, asthma, allergic rhinitis and chronic obstructive pulmonary disease, which affect a large part of the world population, stand out. Due to the lack of studies with *L. alba* on the upper respiratory tract muscles, the present study sought to evaluate the effect of the essential oil of *Lippia alba* (EOLa) and its main constituents, citral and limonene, on the tracheal smooth muscle contractions of Wistar rats.

## 2 Materials and methods

### 2.1 Botanic material

The botanical material was obtained at the UFC experimental farm by Dr. Sergio Horta (experimental farm of the Federal University of Ceará, Brazil). The essential oil extracted from a *Lippia alba* (Mill.) N. E. Brown sample was chemically analyzed in the Laboratory of Natural Products and the Technological Development Park (PADETEC) of the Federal University of Ceará, Brazil, with the following compounds being detected: citral 75.92% [Geraniol (41.81%) and neral (34.11%)], limonene (9.85%), carvone (8.92%), gamma-terpinene (2.05%), cymene (1.02%). As observed in work by Sousa et al. [13].

### 2.2 Substances and solutions

The nutrient solution containing the following composition in mM: NaCl = 136; KCl = 5.0; MgCl_2_ = 0.98; NaH_2_PO_4_ = 0.36; NaHCO_3_ = 11.9; CaCl_2_ = 2.0 and C_6_H_12_O_6_ = 5.5, was maintained at 37 °C and the pH was adjusted to 7.4, remaining in stabilization for 1 h. The EOLa, citral and limonene, were prepared as a solution, diluted directly into Tyrode and Tween (3%). The calcium-free solution or “zero calcium” (0 Ca^2+^) was produced without CaCl_2_ in the Tyrode solution and an addition of 0.2 mM EGTA. All salts and reagents used were of analytical grade and purity obtained from the company Sigma-Aldrich (St. Louis, Missouri, USA). To confirm whether smooth muscle relaxation is dependent on voltage-operated cation channels (VOCCs), BaCl_2_ and nifedipine (1 μM) were used.

### 2.3 Animals and experimental procedures

Male Wistar rats (*Rattus norvegicus*) with a body mass of 200–300 g, obtained from the Central Biotechnology of the Regional University of Cariri-URCA, Brazil, were used. The animals were kept under constant humidity and temperature conditions of 23 ± 2 °C, in a twelve hours light/dark cycle, with access to water and ration *ad libitum*, and were treated according to the Brazilian College of Animal Experimentation (COBEA), Brazil. The study was approved by the Committee on Ethics in the Use of Animals (CEUA), registered under the protocol number: under No. 24/2012.2/2012.

The animals were euthanised in a CO_2_ chamber, followed by the dissection of the trachea, which was sectioned into circular transverse segments of approximately 3 to 4 mm in length, which were then mounted in isolated organ bath tubs with a capacity for 10 mL of modified Tyrode nutrient solution, maintained under continuous aeration by O_2_ bubbling, at 37 °C and pH 7.4 for 60 min. Consequently, the contracting agonists KCl (60 mM) and ACh (10 μM) were added to the organ baths in distinct experiments and followed by a crescent and cumulative addition of the EOLa, citral and limonene separately.

The data were presented as the mean ± S.E.M. and N, where N represents the number of experiments and S.E.M. means the standard error of the mean. The software Sigma Plot 11.0 was used for statistical analysis and the production of graphs. The results considered statistically significant had a null hypothesis probability of less than 5% (p ˂ 0.05). The Analysis of Variance test (*one-way* ANOVA), followed by the *Holm-Sidak* multiple comparisons test when appropriate, were used. For the calculation of the EC_50_, logarithmic interpolation was performed, which was considered as the concentration of the substance which is able to produce 50% of its inhibition or its maximum effect, and the calculations were performed for each experiment.

## 3 Results

**Fig. 1:**
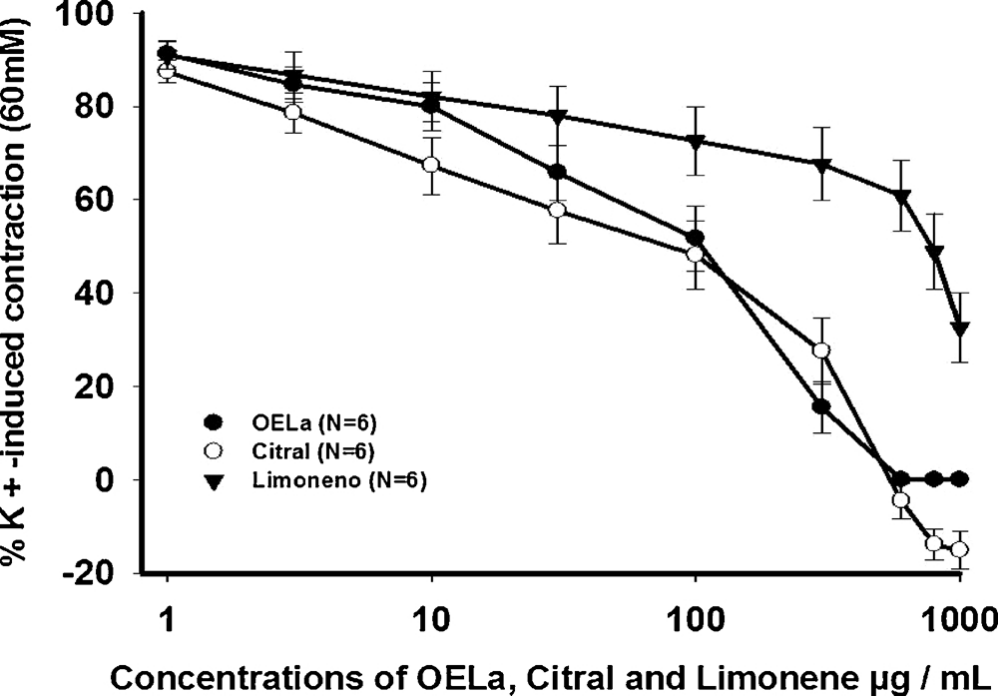
Concentration-effect curve of OELa, citral and limonene (1–1000 μg/mL), under contraction evoked by KCl (60 mM) in trachea isolated from rats. Values are expressed as mean ± SEM, (p < 0.05, one-way ANOVA followed by Holm-Sidak), where N represents the number of experiments.

In the assessment of basal tonus tracheal, the increasing and cumulative concentrations of EOLa, citral and limonene (1–1000 μg/mL), were added to the tracheal rings of Wistar rats at baseline. The results showed that there was no statistically significant relaxant or contracting effect on the basal tonus of the tracheal ring preparations (p ˃ 0.05, *one-way* ANOVA). In preparations pre-contracted with the KCl (60 mM), increasing concentrations of the EOLa, citral and limonene (1–1000 μg/mL), promoted concentration-dependent relaxation, where significant effects were observed in the concentrations ≥30 μg/mL for the EOLa, ≥30 μg/mL for citral and ≥600 μg/mL for limonene, presenting with an EC_50_ of 148 ± 7 μg/mL for the EOLa, 136 ± 7 μg/mL for citral and 581 ± 7 μg/mL for limonene, with these being statistically significant (Fig. 1) (p < 0.001, *one-way* ANOVA followed by *Holm- Sidak*).

**Fig. 2:**
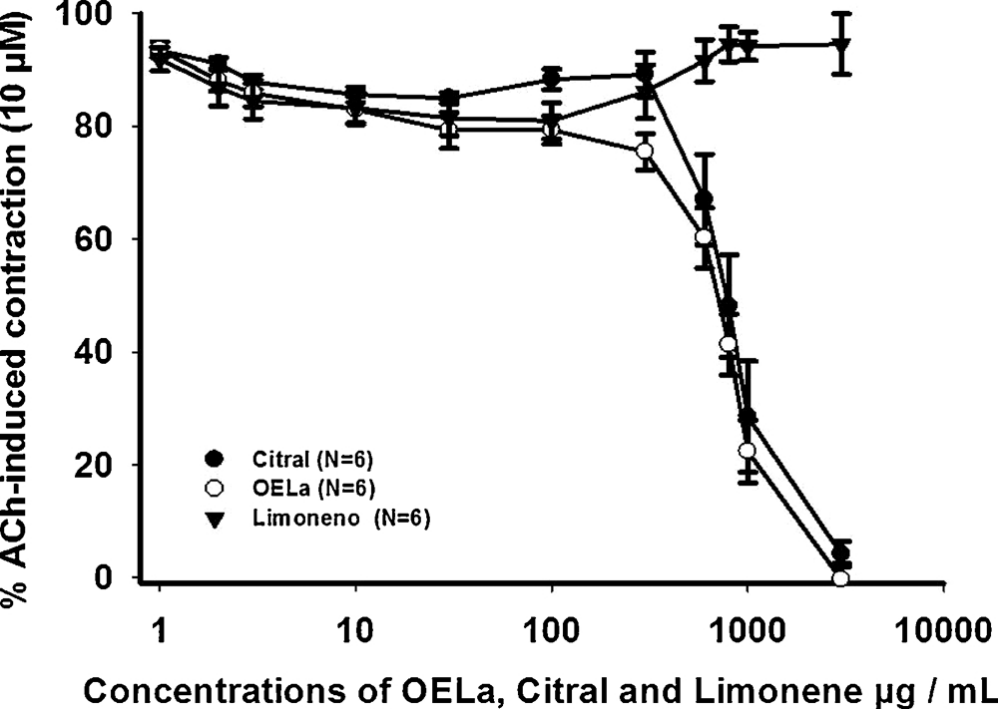
Concentration-effect curve of OELa, citral and limonene (1–3000 μg/mL), in contraction evoked by ACh (10 μM) in trachea isolated from rats. Values are expressed as mean ± S.E.M, (p < 0.05, one-way ANOVA followed by Holm-Sidak), where N represents the number of experiments.

When investigating the activity of the EOLa, citral and limonene (1–3000 μg/mL), on the contractions evoked by ACh (10 μM), there was a myorelaxant activity for EOLa and citral, and its effects were significant in concentrations ≥300 μg/mL for the EOLa and ≥600 μg/mL for citral, presenting with an EC_50_ of 731 ± 5 μg/mL and 795 ± 9 μg/mL for the EOLa and citral, respectively (Fig. 2) (p < 0.001, *one-way* ANOVA, followed by *Holm-sidak*). Limonene was not able to produce a statistically significant myorelaxant effect (p ˃ 0.05, *one-way* ANOVA).

According to the results, we can observe that the EOLa and citral have their most pronounced action in the electromechanical pathway, when comparing its results with the pharmacological route, thus demonstrating that the EOLa and citral act predominantly on VOCCs channels.

To investigate whether this smooth muscle relaxation occurs through L-type calcium channels, we used cumulative BaCl_2_ (1–30 mM) concentrations, which is an ion with selectivity for VOCCs [14].

**Fig. 3:**
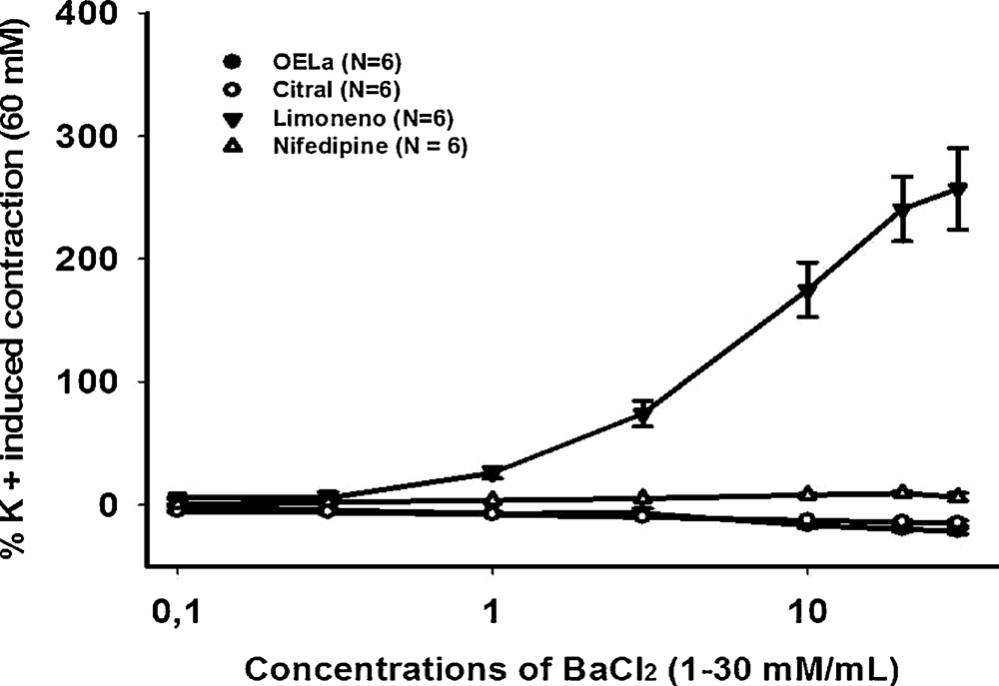
Evaluation of the participation of calcium channels in the relaxation produced by OELa, citral and limonene in isolated rat trachea. Effect of OELa, citral and limonene (1000 μg/mL), in contractions evoked by exogenous BaCl_2_, nifedipine (1 μM) was used as a positive control. Values are expressed as mean ± S.E.M, (p < 0.05, one-way ANOVA followed by Holm-Sidak), where N represents the number of experiments.

In preparations pre-incubated with 1000 μg/mL of the EOLa and citral, it was possible to verify that both agents blocked the influx of BaCl_2_ by the VOCCs, promoting smooth muscle relaxation, with a similar behavior to that of nifedipine (1 μM), this being a voltage-dependent calcium channel blocker. Whereas in preparations induced with 1000 μM limonene, the contraction induced by BaCl_2_ was allowed, demonstrating that limonene does not block L-type calcium channels (Fig. 3).

## 4 Discussion

*L. alba* has a large biological distribution of the species in America, which is based mainly on the chemotypes and their geographic distribution [15] The results demonstrate that the EOLa, citral and limonene promoted relaxation of tracheal smooth muscles from Wistar rats, both in potassium and acetylcholine induced contractions. On the other hand, we did not observe any alteration on muscle tone, this result is relevant as the EOLa and its constituents do not alter the physiology of the tissue, moreover it has already been seen that the EOLa possesses low toxicity [16].

When analyzing the EOLa and its constituents, citral and limonene, over 60 mM KCl evoked contractions, it is observed that the EOLa and citral were able to inhibit tracheal smooth muscle contractility from the concentrations of 30 μg/mL for the EOLa and for citral. In contrast, limonene had a lower effect, whose initial concentration with significance was from 600 μg/mL. This thus suggests that citral is responsible for the myorelaxant activity in the EOLa, since it represents 75% of the EOLa, followed by limonene (9.85%). Antispasmodic and neuronal excitability blocking activities were verified in studies by Blanco et al. [4] and Sousa et al. [13] respectively, these being attributed to the presence of citral. Evi; Im; Smail [17], also attributed the antispasmodic activity of *Cybopongon citratus* (DC) Stapf to citral, this being its major component.

These results suggest that the EOLa and citral have low activity on muscarinic receptors and consequently promote little or no influx of Ca^2+^ through ligand-dependent calcium channels (SOCCs and ROCCs) since they require secondary signaling messengers (IP3) and Diacylglycerol (DAG) that are activated by active ACh [18].

Inhibition of the contractions evoked by BaCl_2_ suggests an interaction of the EOLa and citral with VOCC receptors, which mediate Ca^2+^ influx through channel activation due to changes in membrane voltage. The affinity of the EOLa and citral with VOCCs resembles that of nifedipine, a selective blocker of L-type Ca^2+^ channels [19].

The activation of L-type Ca^2+^ channels occur through high depolarization [20], where these channels are still quite permeable to BaCl_2_. Since the EOLa and citral were able to inhibit contractions evoked by the presence of K^+^ (electromechanical coupling) and blocked contractions evoked by BaCl_2_ dose-response curves, behaving similarly to nifedipine, it is proposed that relaxation of the tracheal smooth musculature mediated by the EOLa and citral occurs via electromechanical coupling, probably due to a blockade of L-type VOCCs [21]. Limonene on the other hand, although it had a relaxing effect on the preparations exposed to K^+^, showed no significant effect in the presence of the ACh agonist. In addition, limonene was unable to inhibit contractions evoked by consecutive BaCl_2_ (1–30 mM) concentrations, suggesting that its action is not involved with pharmaco-mechanical coupling or with L-type channels. However, limonene activity may still be linked to VOCCs, but not to those which are L-type.

According to the present study, the EOLa and its main compounds, citral and limonene, found in the *Lippia alba* species, showed a relaxing effect on isolated trachea from rats. However, further research is required since the results suggest the possibility of the appearance of new relaxing substances.

The study demonstrated that the EOLa and its major compound citral have an antispasmodic effect over tracheal smooth muscle from Wistar rats, although citral is more significant. This effect has been attributed to the blockade of L-type VOCC channels. The data obtained in this study demonstrate that the EOLa and citral have great pharmacological potential for use in respiratory diseases.

## Conflict of interest

The authors declare no conflict of interests.
